# Fitness promotion in college: the relationships among students’ perceived physical literacy, knowledge, and physical fitness

**DOI:** 10.3389/fpsyg.2024.1305121

**Published:** 2024-04-25

**Authors:** Bin Long, Yang Liu, Senlin Chen, Yiwu Yang, Suna Tang, Jimei Yang, Yuhuai Wang

**Affiliations:** ^1^School of Sports Training, Wuhan Sports University, Wuhan, China; ^2^School of Physical Education, Wuhan University of Technology, Wuhan, China; ^3^School of Kinesiology, Louisiana State University, Baton Rouge, LA, United States

**Keywords:** fitness assessment, fitness promotion, physical activity, physical literacy, undergraduate students

## Abstract

**Purpose:**

The purpose of this study was to examine the relationships among perceived physical literacy (PPL), knowledge of physical activity and fitness (PAF knowledge), and physical fitness.

**Methods:**

Undergraduates (*N* = 968, female = 414; *M*_age_ = 18.64) from a public university in central China completed a simplified Chinese version of the PPL instrument, an online test for PAF knowledge, and seven health-related physical fitness tests. The PPL includes three dimensions: (a) *confidence and physical competence*, (b) *motivation*, and (c) *interaction with the environment*. The fitness tests measured lung capacity, body mass index (BMI), and performances in 800 (female)/1000 (male) meters run, 50 meters dash (50 M), sit-up (female) / pull-up (male), standing long jump (SLJ), and sit-and-reach.

**Results:**

PPL and its dimensions significantly predicted six fitness test performances both in male (*β*: −0.42 – 0.37; *p* < 0.01–0.05; *R*^2^: 0.01–0.13) and female (*β*: −0.59 – 0.49; *p* < 0.01–0.05; *R*^2^: 0.03–0.13) students. PAF knowledge (*β*: −0.17 – 0.18; *p* < 0.01–0.05; *R*^2^: 0.01–0.05) significantly predicted BMI (males) and performances in 50 M (females) and SLJ (females) tests.

**Conclusion:**

To support college students’ fitness development and maintenance, tailored physical activity and fitness promotion programs are needed to strengthen students’ PPL and PAF knowledge.

## Introduction

College is an important period for young adults to acquire specialized higher education needed for the workforce and learn how to live independently. College students deal with multiple responsibilities from which they encounter stressors and many develop mental health disorders, making them a vulnerable population, especially during the COVID-19 pandemic (Pat-Horenczyk et al., 2022; Wang and Liu, 2022). To this population, achieving and maintaining an adequate level of physical fitness is crucial for these young adults’ overall health, well-being, and daily functioning in college (Tong et al., 2021). However, most college students struggle to demonstrate sufficient levels of physical activity participation, hampering their physical fitness development and health outcomes. For example, prior to the COVID-19 pandemic, only 30% of college students met the physical activity guidelines (Hallal et al., 2012; Song et al., 2013; Arias-Palencia et al., 2015). During the pandemic, approximately 82% of Chinese students in the middle and late adolescence stages demonstrated fair or poor levels of physical fitness (Bureau of Sports, Hygiene, and Arts Education, Ministry of Education of the People’s Republic of China, 2021). In China, college students are required to complete the national fitness tests each school year and nearly all colleges and universities offer elective physical activity courses for students. A fitness investigation conducted prior to the COVID-19 pandemic involving over 150,000 college students reported an average score of 68.8% for their fitness (Sports Bureau of Zhejiang Province, General Administration of Sport of China, 2015). College students’ fitness status might have worsened during the pandemic. Importantly, despite the mandatory fitness testing in Chinese universities and colleges, there seems to be a disconnect between fitness assessment and fitness promotion efforts (Bureau of Sports, Hygiene, and Arts Education, Ministry of Education of the People’s Republic of China, 2021). Rarely have colleges and universities put forth deliberate plans to improve students’ fitness based on the fitness assessment results. Further, to effectively promote physical activity and fitness during college years, it is critically important to identify and address influential correlates and determinants of physical activity and fitness.

Physical literacy (PL) offers a unique and useful perspective for lifelong physical activity and fitness promotion. PL has been widely discussed in numerous countries due to its relevance to physical education, youth development, and physical activity and health promotion (Liu and Chen, 2021). The International Physical Literacy Association (IPLA) describes PL as a concept that addresses an individual’s knowledge and understanding for physical activity and health, motor skills, physical competence, and lifelong physical activity participation. Although PL has been conceptualized divergently across perspectives and contexts (Roetert and Jefferies, 2014; Society of Health and Physical Educators (SHAPE) America, 2014; Lounsbery and McKenzie, 2015; The Aspen Institute, 2015), there is a consensus that the ultimate goal of PL development is to adopt physically active lifestyles throughout the entire lifespan (Whitehead, 2013; Canadian Sport for Life, 2015). Achieving sufficient levels of PL has been recognized as a precursor of lifelong physical activity participation and health-enhancing physical fitness (Liu and Chen, 2022).

While most existing research has focused on PL and PL development, fewer studies have emphasized students’ perceived PL (PPL). PPL refers to a person’s self-perception of their overall PL as well as PL attributes across physical, behavioral, cognitive, and affective domains. PPL resembles perceived competence, capturing a person’s self-concept or self-efficacy beliefs about their PL capabilities across domains and attributes. Numerous prior studies have made the case for perceived competence and self-efficacy for physical activity promotion (Babic et al., 2014), as they are usually considered proxy or determinants of behaviors (i.e., initiation, adherence), performance, and achievement. Likewise, PPL could be an influential correlate or determinant of physical activity and fitness. However, rarely has prior research empirically examined PPL (along with physical activity and fitness [PAF] knowledge) for PL development, physical activity, and fitness promotion. As exceptions, several studies shed light on the relationships between PL and physical activity (Yan et al., 2022), between PAF knowledge and physical activity (Thompson and Hannon, 2012; Chen et al., 2017; Liu and Chen, 2020), and between physical activity and physical fitness (Malina, 2001). Yet, all of these prior studies focused on pre-college student populations, and relevant studies on college students are non-existent, warranting future research. College students represent a vulnerable population and their physical fitness level plays a critically important role in their health, well-being, and college life overall.

Given the aforementioned discussion, this study is to examine the relationships among PPL, PAF knowledge, and health-related physical fitness. The study is centered on addressing three research questions: (a) To what extend do PPL and its dimensions associate with PAF knowledge? (b) To what extent do PPL and its dimensions associate with physical fitness? and (c) to what extent does PAF knowledge associate with physical fitness in college students.

## Methods

### Setting and participants

This study recruited 1,219 college students enrolled in physical activity courses (female = 486; *M*_age_ = 18.79, *SD* = 1.11) at a major public university in central China. At the time of data collection, the university enrolled 36,822 undergraduate students. All students were required to take and pass at least four physical activity courses for graduation. Physical activity classes (100 min per lesson) were offered once per week. The contents of the courses were diverse with 20 different sports and physical activities being offered in a semester. The participants were informed of the research purpose, procedures, and their rights to accept/decline their participation or withdraw at any time from the study. The study was approved by the first author’s university’s human subject research ethics committee. Written participants’ consent was secured before data collection.

### Variables and measures

The participants completed an online survey to measure PPL, a written test to measure knowledge of sport and fitness, and completed the annual physical fitness tests. These variables and measures are described below.

#### Perceived physical literacy

PPL was assessed using the simplified Chinese version of the validated Perceived Physical Literacy (PPL) Instrument (construct validity: *χ*^2^ = 1.32, RMSEA = 0.03, AGFI = 0.96, NFI = 0.97, CFI = 0.99, TLI = 0.99; reliability: Cronbach *α*: 0.79–0.83; Ma et al., 2020a). The instrument has eight items capturing three PPL dimensions: (a) *confidence and physical competence*, (b) *motivation*, and (c) *interaction with the environment*. *Confidence and physical competence* refers to the degree to which individuals perceive their holistic capabilities of engaging in various physical activities. *Motivation* refers to the degree to which individuals perceive their reasons, interests, and knowledge concerning the initiation and/or maintenance of physically active lifestyles. *Interaction with the environment* refers to the degree to which individuals perceive their skills in socializing with others (Whitehead, 2001; Ma et al., 2020a). Respondents rated each item on a five-point Likert scale, ranging from 5 = “strongly agree” to 1 = “strongly disagree.” For example, one question capturing *confidence and physical competence* is phrased as “I possess adequate fundamental movement skills.” The aggregated total score of PPL and scores for each PPL dimension were calculated for data analysis. The validity and reliability of the PPL instrument were acceptable with the present sample (as reported in the result section).

#### Physical fitness

Seven physical fitness tests (in simplified Chinese) developed by LingKang Electronic Technology Development Co., Ltd. were conducted by the physical activity teachers. The fitness tests had been endorsed by the Ministry of Education of China and subsequently by all colleges and universities in China to assess college students’ health related physical fitness achievement. The seven fitness tests included (1) lung capacity (in mml) using a spirometer in pulmonary function test, (2) body mass index (BMI) converted from body weight and height data as measured using a weight scale and stadiometer, (3) 800 (female; 800 M) / 1,000 (male; 1,000 M) meters run (in second), (4) 50 meters dash (50 M) at maximum effort, (5) 1-min sit-up (female) / pull-up (male) tests, (6) standing long jump (SLJ; in centimeter), and (7) sit-and-reach (in centimeter). All fitness tests were administered by trained physical activity teachers who followed the fitness testing manual.

#### Physical activity and fitness knowledge

The PAF knowledge test (in simplified Chinese) captures students’ knowledge and understanding about 19 different physical activities (e.g., sports) including their history and characteristics, basic rules, movement skills involved, and strategies for improving performances and/or fitness; as well as concerning how to maintain active living and health-enhancing fitness through participation in these physical activities. The PAF knowledge test was administered online through the intelligent testing platform developed by the Lepao Sports Internet (Wuhan) Co., Ltd. The written test (100 points maximum possible) consisted of 20 questions each with one correct answer (2 points for each correct response, 40 points subtotal), 20 questions each with multiple correct answers (2 points for each correct response, 40 points subtotal), and 20 true/false questions (1 point for each correct response, 20 points subtotal). The test questions were randomly selected from the pool of 5,603 questions at each time of administering the test. The test bank was established by a content expert committee consisting of 16 physical activity instructors with an average of 12 years of teaching experience. To improve the test validity, 16 additional physical activity instructors proofread and refined all the questions. One example of a true/false question is phrased as, “physical activity can increase the quantity of lymph cells, and as a result can promote immunology.” An example multiple choice question is phrased as “to develop aerobic capacity, you can do long-distance running at moderate intensity, and maintain a heart rate of beats/min;” with “90–120,” “110–130,” “130–150,” and “190–210” being the choices. Responses to the test items were automatically scored in reference to the answer key and the correct percent score was obtained by dividing the total points earned by 100.

### Data collection

Two rounds of online surveys collecting PPL data were conducted in late December 2020. Before the survey, all participating teachers received adequate training on how to conduct the assessments and collect data following standardized procedures. The first survey collected 251 responses, which were used to examine the reliability and validity of the PPL instrument. The second PPL survey yielded 968 responses, which were analyzed to address the research purpose. The PPL survey was created by a research assistant on Sojump, a survey application for making online surveys. The survey link was shared with all participating teachers (i.e., data collectors) at least 3 days before data collection. At the beginning of each physical activity class, student participants were shared with the QR code to access and then complete the PPL survey on their smartphones. They were asked to complete the survey within 10 min.

The PAF knowledge test was administered at a similar time as the PPL survey. One week before the test, the participants were informed of the test date, followed by a reminder sent by the physical activity instructors 1 day before the test. The test time was not fixed during the two testing days. Participants completed the test online within the allotted 60 min. Each participant was instructed to take the test independently and was allowed to take the test for two trials, of which the better score was recorded as the final test score.

The seven physical fitness tests were administered by trained physical activity instructors on multiple weekends in December 2020. Tests across all fitness items were available to the participants from 8 a.m. to 5 p.m. in the open stadium. Before and after the fitness tests, participants were organized by three physical activity instructors to receive sufficient warm-up and cool-down, respectively. Participants were told there was no testing sequence, but 50 M and long-distance runs should be completed on different days and be the last items on each testing day to avoid effort interferences across tests. It took on average 18 min per participant to complete all fitness tests, where lung capacity (in groups of 6), 50 M (in groups of 6), long-distance running (in groups of 70), and sit-up (in groups of 5) were measured.

### Data processing and analyses

Data for the physical fitness and PAF knowledge were downloaded directly from the Lepao intelligent platform and saved in Microsoft Excel. Responses to the PPL items in the second survey were aggregated by three dimensions (i.e., *confidence and physical competence*, *motivation*, and *interaction with the environment*). All data across the tests were matched and organized at the individual level. Preliminary data processing was conducted using M ± 3*SD to screen the data of each variable for outliers. Given the minimal data missingness, the pair-wise deletion approach was adopted for treating missing values in all data analysis.

PPL data collected at the first survey were used to examine the validity and reliability of the instrument using confirmatory factor analysis (CFA) and Cronbach’s *α*. PPL data collected from the second survey were used to address the research purpose. Descriptive analyses were performed for physical fitness, PAF knowledge, and PPL (e.g., mean, standard deviation) by gender and grade. Before inferential analyses, raw data were standardized to obtain Z scores. Path analyses were applied by gender to examine (a) the associations between three PPL dimensions (predictors) and seven physical fitness test results; (b) the associations between PPL (predictor) and seven physical fitness results; (c) the associations between PPL (predictor) and PAF knowledge; (d) the associations between three PPL dimensions and PAF knowledge; and (e) the associations between PAF knowledge and seven physical fitness tests. In all path analyses, age was entered as a covariate, due to its potential moderating effect. All statistical analyses either were performed using R 4.3.1. The significant level was set at 0.05.

## Results

### Construct validity of PPL instrument and descriptive analyses

The results of CFA examining the data collected from the first round survey showed that the PPL instrument had acceptable validity (*χ*^2^ = 2.50, RMSEA = 0.08, AGFI = 0.92, GFI = 0.96, SRMR = 0.04, CFI = 0.97, TLI = 0.96) and reliability (Cronbach *α*: 0.73–0.92). The descriptive characteristics of PAF knowledge, PPL (and its three PPL dimensions), and seven physical fitness test results are displayed in Table 1. There are notable gender differences in PAF knowledge, two PPL dimensions, PPL, 50 M, sit-and-reach, SLJ, lung capacity, and BMI.

**Table 1:** Descriptive analysis result by gender.

Variables	Gender						Overall		
	Male			Female					
	Mean	*N*	SD	Mean	*N*	SD	Mean	*N*	SD
Knowledge^***^	94.68	519	8.22	96.64	395	5.30	95.53	914	7.17
PPL D1^**^	11.30	554	2.33	11.75	414	1.98	11.49	968	2.19
PPL D2	12.82	553	1.66	12.98	413	1.40	12.89	966	1.56
PPL D3^**^	7.35	554	1.79	7.66	414	1.65	7.48	968	1.74
PPL Total^**^	31.48	553	4.66	32.39	413	4.07	31.87	966	4.44
800 M (Female)	---	---	---	242.41	368	23.12	---	---	---
1,000 M (Male)	250.15	493	28.73	---	---	---			
50 M^***^	8.05	497	0.67	9.69	368	0.66	8.75	865	1.05
SU	---	---	---	36.01	368	9.03	---	---	---
PU	4.60	487	4.42	---	---	---			
Sit-and-reach^***^	14.99	495	8.27	18.42	366	6.30	16.45	861	7.68
SLJ^***^	214.74	497	21.94	161.82	369	16.34	192.19	866	32.79
Lung Capacity^***^	4553.30	497	798.81	3182.09	369	586.47	3969.03	866	986.17
BMI^***^	22.15	496	3.99	20.64	369	3.01	21.50	865	3.68

Knowledge, knowledge of physical activity and fitness; PPL D1, The first dimension of PPL; PPL D2, The second dimension of PPL; PPL D3, The third dimension of PPL; PPL total, The total score of PPL instrument; 800 M, 800 meters long distance running; 1,000 M, 1000 meters long distance running; SU, sit up; PU, push up; SLJ, standing long jump.

### Correlational strength among variables

The correlational analysis showed that PAF knowledge (*r*: 0.19–0.20) and PPL (|*r*|: 0.11–0.23) as well as its dimensions (|*r*|: 0.11–0.28) significantly associated with fitness testing results; and that PAF knowledge and PPL (and its dimensions) did not correlate with each other (Table 2). We subsequently separated the path analysis by gender.

**Table 2:** Correlational matrix of both genders.

Female/Male	1	2	3	4	5	6	7	8	9	10	11	12
KG	1	0.07	0.08	0.05	0.08	−0.08	0.07	0.04	0.20**	0.19**	−0.02	−0.01
D1	−0.02	1	0.49**	0.52**	0.87**	−0.28**	0.19**	0.13*	0.15**	−0.22**	0.08	−0.18**
D2	0.02	0.44**	1	0.40**	0.75**	−0.12*	0.16**	0.14**	0.03	−0.07	0.07	0.06
D3	0.02	0.53**	0.42**	1	0.80**	−0.13*	0.13*	0.17**	−0.02	−0.04	0.11*	0
PPL	0.01	0.86**	0.74**	0.80**	1	−0.23**	0.20**	0.17**	0.08	−0.15**	0.11*	−0.07
1000/800 M	0.03	−0.30**	−0.16**	−0.14**	−0.25**	1	−0.17**	−0.07	−0.31**	0.42**	−0.10	0.27**
SU/PU	0	0.22**	0.10*	0.01	0.15**	−0.38**	1	0.15**	0.18**	−0.15**	0.03	−0.07
SR	0.05	0.07	0.05	0.04	0.07	−0.09	0.08	1	0.13*	−0.16**	0.10	−0.02
SLJ	0.05	0.30**	0.12**	0.14**	0.25**	−0.41**	0.30**	0.13**	1	−0.50**	0.15**	−0.17**
50 M	−0.06	−0.29**	−0.09	−0.08	−0.21**	0.45**	−0.28**	0.01	−0.43**	1	−0.14**	0.16**
LC	−0.03	0.06	0.08	0.14**	0.11*	−0.01	−0.12**	0.07	−0.03	−0.01	1	0.16**
BMI	−0.09*	−0.15**	−0.06	0.09*	−0.06	0.34**	−0.30**	0.01	−0.26**	0.28**	0.25**	1

KG, knowledge of physical activity and fitness; D1, The first dimension of PPL; D2, The second dimension of PPL; D3, The third dimension of PPL; PPL, The total score of PPL instrument; 800 M, 800 meters long distance running; 1,000 M, 1000 meters long distance running; SU, sit up; PU, push up; Jump, standing long jump; SR, sit and reach; SLJ, standing long jump; LC, lung capacity; * means significant level of 0.05; ** means significant level of 0.01.

### The predictive effects of PPL and PAF knowledge on physical fitness in males

**Figure 1:**
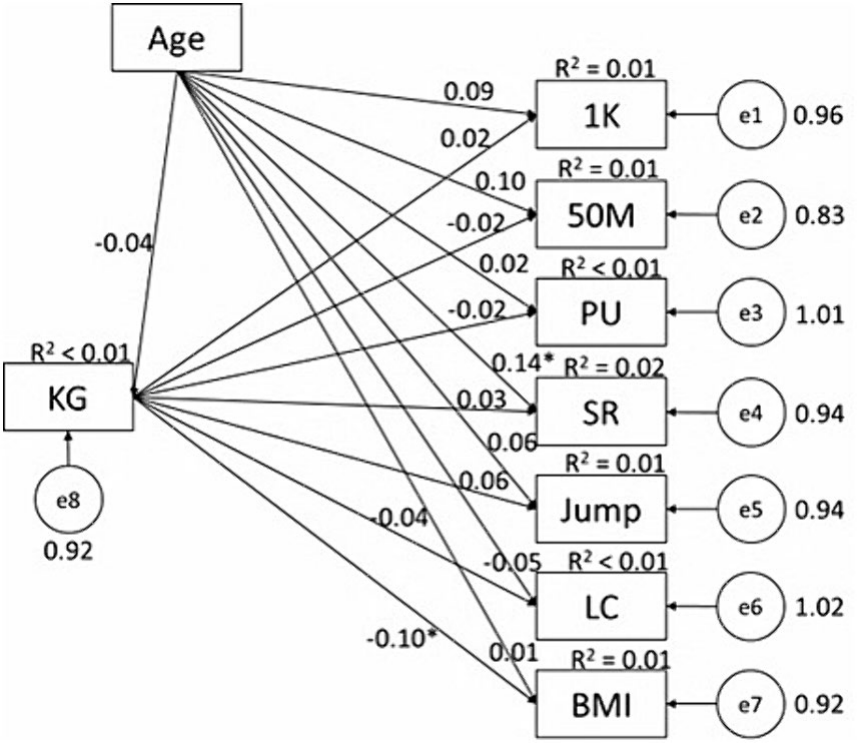
Associations between PAF knowledge and health-related physical fitness variables in male college students. KG, PAF knowledge; PU, pull-up; SR, sit-and-reach; Jump, standing long jump; LC, Lung capacity.

**Figure 2:**
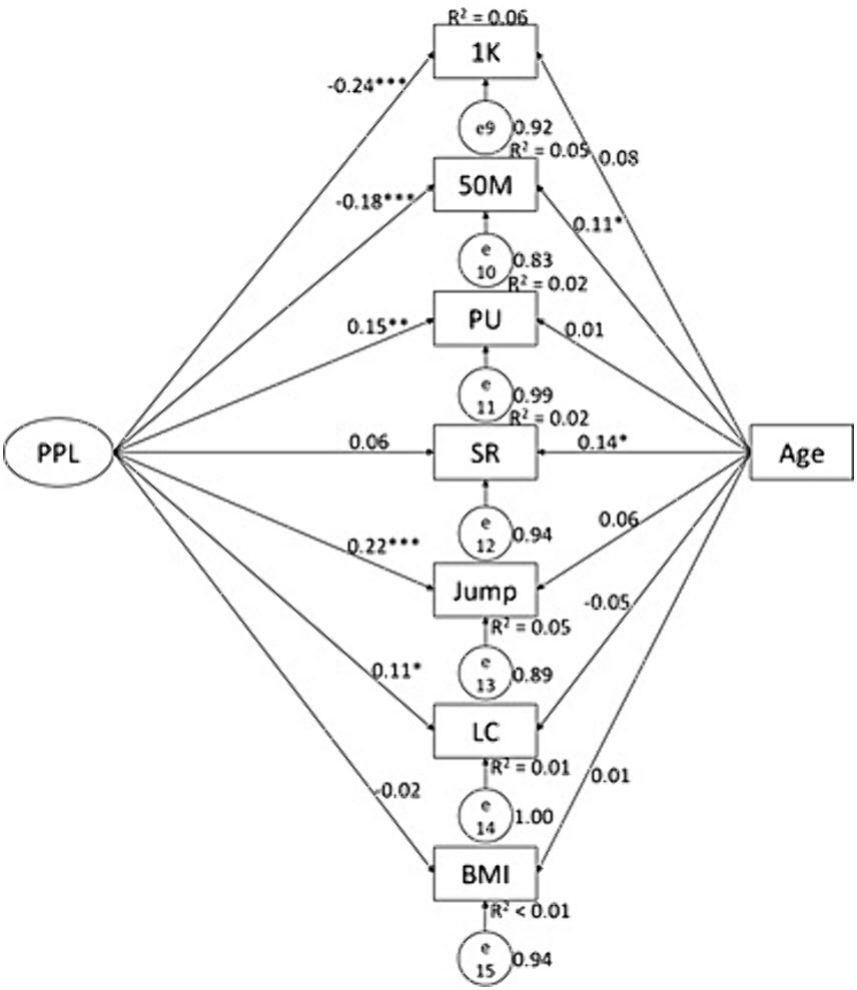
Associations between PPL and health-related physical fitness variables in male college students. 1 K, 1,000 meters running; PU, pull-up; SR, sit-and-reach; Jump, standing long jump; LC, Lung capacity.

**Figure 3:**
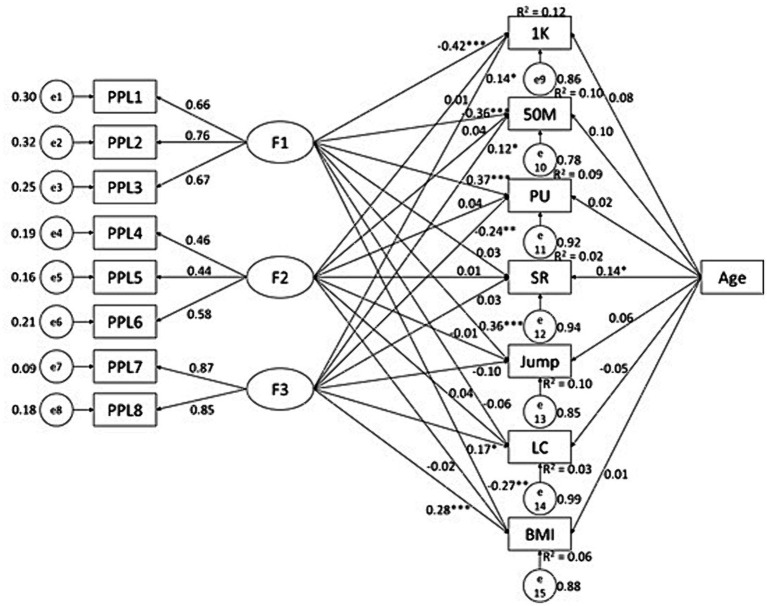
Associations between PPL dimensions and health-related physical fitness variables in male college students. F1, F2, and F3 are three dimensions of PPL; 1 K, 1,000 meters running; PU, pull-up; SR, sit-and-reach; Jump, standing long jump; LC, Lung capacity.

**Figure 4:**
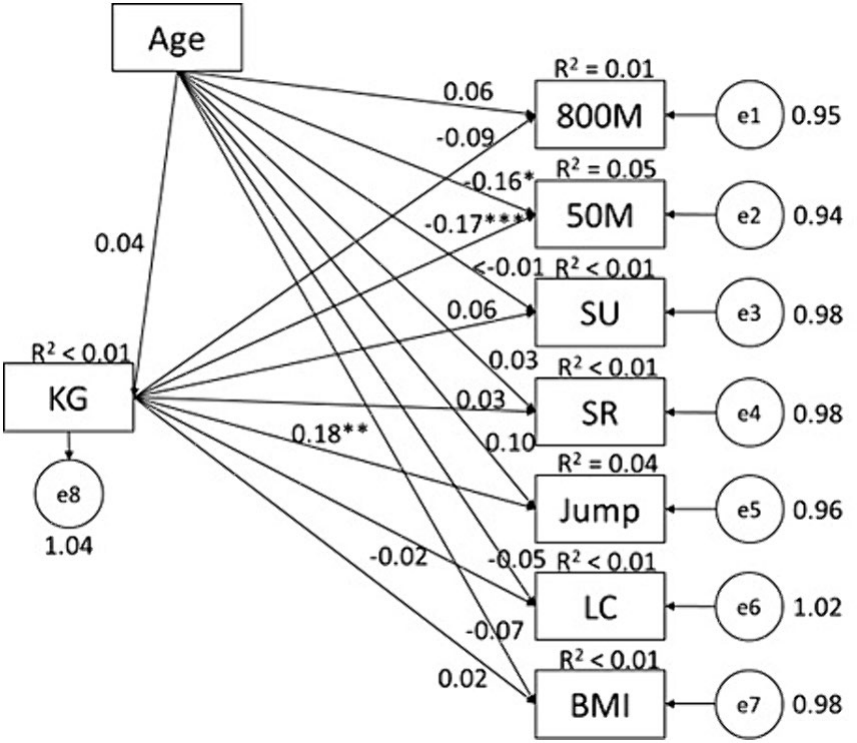
Associations between PPL and health-related physical fitness variables in female college students. SU, sit-up; SR, sit-and-reach; Jump, standing long jump; LC, Lung capacity.

**Figure 5:**
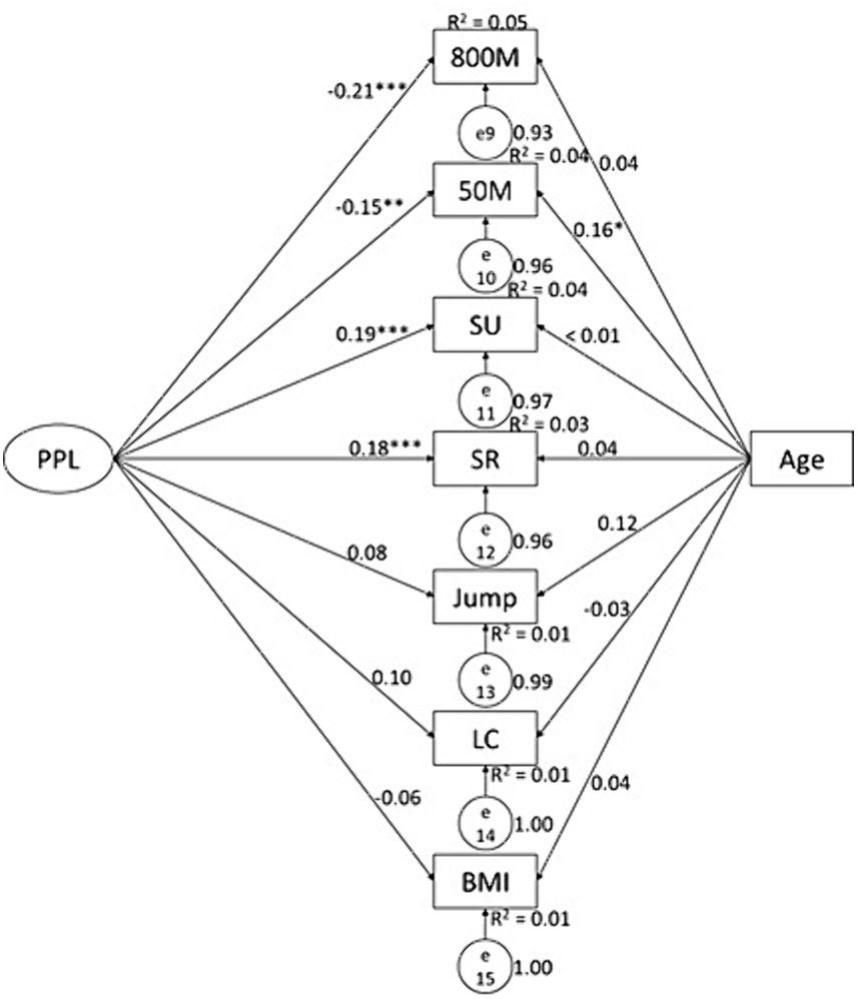
Associations between PAF knowledge and health-related physical fitness variables in female college students. KG, PAF knowledge; SU, sit-up; SR, sit-and-reach; Jump, standing long jump; LC, Lung capacity.

**Figure 6:**
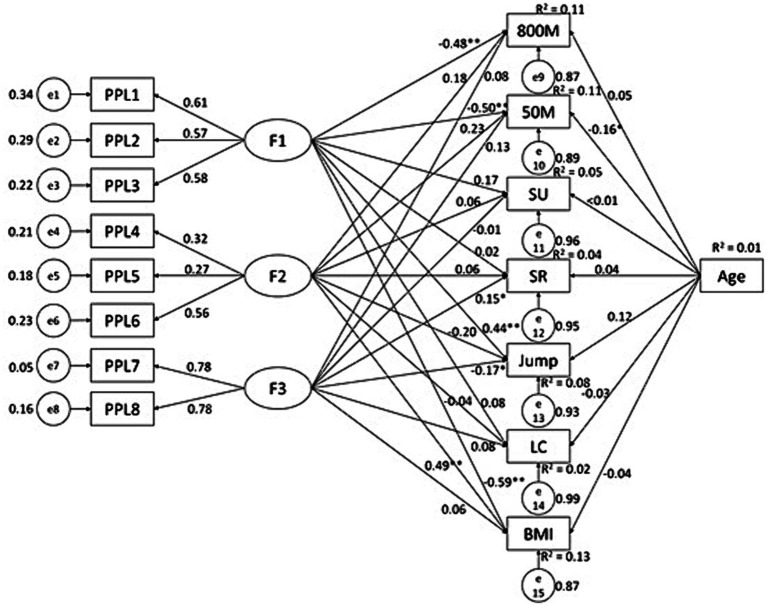
Associations between PPL dimensions and health-related physical fitness variables in female college students. F1, F2, and F3 are three dimensions of PPL; SU, sit-up; SR, sit-and-reach; Jump, standing long jump; LC, Lung capacity.

In male participants, we found some interesting results patterns. PAF knowledge negatively predicted BMI (*β* = −0.10, *p* < 0.05, *R*^2^ = 0.01) (Figure 1); while PPL significantly predicted 1,000 M (*β* = −0.24, *p* < 0.01, *R*^2^ = 0.06), 50 M (*β* = −0.18, *p* < 0.01, *R*^2^ = 0.05), pull-up (*β* = 0.15, *p* < 0.01, *R*^2^ = 0.02), SLJ (*β* = 0.22, *p* < 0.01, *R*^2^ = 0.05), and lung capacity (*β* = 0.11, *p* < 0.05, *R*^2^ = 0.01) (Figure 2); with all predictive relationships being conceptually desirable. Of the PPL dimensions (Figure 3), *confidence and physical competence* significantly predicted testing results of 1,000 M (*β* = −0.42, *p* < 0.01, *R*^2^ = 0.13), 50 M (*β* = −0.36, *p* < 0.01, *R*^2^ = 0.09), pull-up (*β* = 0.37, *p* < 0.01, *R*^2^ = 0.09), SLJ (*β* = 0.37, *p* < 0.01, *R*^2^ = 0.10), and BMI (*β* = −0.27, *p* < 0.01, *R*^2^ = 0.07); while *interaction with the environment* significantly predicted testing result of LC (*β* = 0.17, *p* < 0.05, *R*^2^ = 0.02). Age as covariate significantly predicted results of 50 M (*β* = 0.11, *p* < 0.05; Figure 2) and SR (*β* = 0.14, *p* < 0.05; Figures 1–3) in male students; and significantly predicted 50 M results (*β* = −0.16, *p* < 0.05; Figures 4–6) in female students.

### The predictive effects of PPL and PAF knowledge on physical fitness in females

Data from female students showed some similar patterns but also with differences. PAF knowledge predicted results of 50 M (*β* = −0.17, *p* < 0.01, *R*^2^ = 0.05) and SLJ (*β* = 0.18, *p* < 0.01, *R*^2^ = 0.04) (Figure 4); while PPL predicted results of 800 M (*β* = −0.21, *p* < 0.01, *R*^2^ = 0.05), 50 M (*β* = −0.15, *p* < 0.01, *R*^2^ = 0.04), sit-up (*β* = 0.19, *p* < 0.01, *R*^2^ = 0.04), and sit-and-reach (*β* = 0.18, *p* < 0.01, *R*^2^ = 0.03) (Figure 5). Of the PPL dimensions, *confidence and physical competence* predicted testing results of 800 M (*β* = −0.48, *p* < 0.01, *R*^2^ = 0.11), 50 M (*β* = −0.50, *p* < 0.01, *R*^2^ = 0.11), SLJ (*β* = 0.44, *p* < 0.01, *R*^2^ = 0.08), and BMI (*β* = −0.59, *p* < 0.01, *R*^2^ = 0.13); and *interaction with the environment* predicted sit-and-reach (*β* = 0.15, *p* < 0.05, *R*^2^ = 0.04).

In addition to the conceptually desirable results reported above, we also observed several undesirable results. *Interaction with the environment* significantly predicted the testing results of 1,000 M (*β* = 0.14, *p* < 0.05, *R*^2^ = 0.13), 50 M (*β* = 0.12, *p* < 0.05, *R*^2^ = 0.09), pull-up (*β* = −0.24, *p* < 0.01, *R*^2^ = 0.09) and BMI (*β* = 0.28, *p* < 0.01, *R*^2^ = 0.06) in males. In the female sample, *motivation* predicted BMI (*β* = 0.49, *p* < 0.01, *R*^2^ = 0.13) and *interaction with the environment* predicted SLJ (*β* = −0.17, *p* < 0.05, *R*^2^ = 0.08). These results indicate that higher scores in several PPL dimensions were associated with lower fitness performance.

## Discussion

This study examined college students’ PPL (and its three dimensions), PAF knowledge, and health-related physical fitness, as well as their relationships. We identified some desirable predictive relationships of PPL and PAF knowledge with physical fitness. We also observed several undesirable predictive relationships of PPL dimensions with certain fitness testing results. The findings are discussed in the following.

PPL and its three dimensions as demonstrated by the participants were slightly higher than previously reported (Ma et al., 2020a). The participants scored very high on the PAF knowledge test (95.53 on average) showing a generally solid understanding of physical activities (e.g., across many sports and games) and its application to enhance fitness. PAF knowledge also showed significant gender difference favoring female students over male students, which aligns with the findings reported in Hunter et al. (2014) and Knox et al. (2015). Additionally, PPL and its two dimensions (i.e., *confidence and physical competence* and *interaction with the environment*) also showed significant gender differences, all favoring female students. This result is inconsistent with previous studies (Ma et al., 2020b; Yan et al., 2022; López-Gil et al., 2023). Future research should further examine the gender differences in PPL and PAF knowledge among college students.

One main finding of the study demonstrated that PAF knowledge is associated with better achievements in 50 M (females), SLJ (females), and BMI (males). This finding is consistent with previous studies. For example, Vaara et al. (2019) reported that young adult men were more knowledgeable about physical activity and fitness, and they were more likely to be physically active and demonstrated higher cardiorespiratory and muscular fitness (e.g., SLJ and sit-up) than their female counterparts. Additionally, possessing more PAF knowledge was found to correlate with better overall health for both males and females (Knox et al., 2015). The identified associations between PAF knowledge and health-related physical fitness are informative to college physical activity education programs, which are recommended to educate students about PAF knowledge and encourage them to regularly participate in physical activities (Thompson and Hannon, 2012; Hunter et al., 2014; Knox et al., 2015; Campos et al., 2019). Valuable PAF knowledge for college students may include but is not limited to, background of various physical activities, the relationship between being physically active/fit and health, selection of a physical activity (e.g., sport), and organization of a sound exercise program.

To improve performances in 50 M and SLJ, female students need to become more knowledgeable about “what to do and how to do,” concerning the training that improves their speed, strength, and power. On the university campus, the available sports and/or exercises for core and lower body power were relatively abundant, including jogging, ball games (e.g., table tennis, volleyball, and badminton), and equipment workouts. For male students, being more knowledgeable about PAF was found to be associated with having a healthier BMI. This finding is meaningful as male college students usually have a higher prevalence of overweight and obesity than their female counterparts (Chen et al., 2020; Bailey et al., 2022), and improving their PAF knowledge could help them achieve a healthier body composition and reduce the obesity risk. Quality physical activity programs should incorporate more knowledge content and educate students to acquire sufficient PAF knowledge to help them understand theories and strategies related to weight control (e.g., energy balance through regular physical activity participation and a healthy diet).

The study further examined the predictive relationship of PPL and its three dimensions with health-related physical fitness, which yielded both conceptually desirable and undesirable findings. We found that college students with higher PPL were more likely to have better performances in 1000 M (males), 800 M (females), 50 M (both genders), pull-up (males), sit-up (females), sit-and-reach (females), SLJ (both genders), and lung capacity (males). For PPL dimensions, being more confident and physically competent predicted higher performances in 1000 M (males), 800 M (females), 50 M (both genders), pull-up (males), SLJ (both genders), and BMI (both genders); and interacting with the environment also predicted better performances in sit-and-reach (females) and lung capacity (males). These findings are viewed as the most promising messages from this study, and are consistent with prior research that suggested the positive relationship between PPL and fitness outcomes such as BMI (Ma et al., 2020b) and aerobic capacity (Nezondet et al., 2023). Other studies further found that PL, not PPL, could facilitate positive health outcomes (Giblin et al., 2014; Ma et al., 2020b). Nevertheless, physically literate individuals through frequent exposure to and participation in sports, games, exercises, and other physical activities usually build adequate physical competence through learning skills, improving performances, enhancing conditioning, and overcoming challenges. The improved physical literacy will ultimately improve their PPL and reinforce their confidence for living active and healthy lifestyles.

Despite the desirable findings discussed above, we also note several counter-intuitive findings. For female students, higher *motivation* was associated with higher BMI. For male students, interacting with the environment predicted lower performances in 1000 M, 50 M, pull-up, and BMI. These results need to be further examined in future research. The relationship between PPL dimensions and individual fitness testing results may be more complex than expected. Measurement error or inaccurate self-perception of PL dimensions could be plausible reasons for these results.

In summary, PAF knowledge and PPL (and its dimensions) deserve to be placed in the center stage for promoting college students’ fitness. Knowing what to do and how and when to move is pivotal for students to adopting active lifestyles (Ennis, 2015) and fitness (Whitehead, 2007; Hunter et al., 2014; Knox et al., 2015; Campos et al., 2019; Vaara et al., 2019; Bailey et al., 2022). Adequate levels of PL contribute to lifelong physical activity participation and health-enhancing fitness achievement (Cairney et al., 2019). Not only does PL matter to physical activity and fitness, but prior research that focused on older adolescents also found that PPL positively correlated with physical activity behaviors (Choi et al., 2018; Gandrieau et al., 2023; Oztürk et al., 2023); and that physical activity mediated the association between PPL and physical fitness (Yan et al., 2022; Nezondet et al., 2023). Emerging adults and young adults in universities and colleges are at the critical stage of fostering voluntary health-enhancing physical activity behaviors and fitness. Physical activity interventions and fitness/wellness workshops in and out of college physical activity programs are in urgent need to promote physical fitness.

To our understanding, this is the first study examining the association of physical fitness with PPL (and its dimensions) and PAF knowledge among college students. Additionally, the study has several notable strengths (i.e., large sample size, valid assessments, and robust data collection; Čehovin et al., 2023; Dzakadzie and Quansah, 2023) and limitations. One limitation is that PPL was measured using a self-reported instrument (i.e., PPL instrument). Despite the inherent limitation of all self-report measures, it would be difficult to assess a person’s self-perception using non-self-report methods. The other limitation of the study is the correlational research design. Cause-effect relationships may not be inferred from our findings. The participants were recruited from one public university dominated by the Han ethnicity. Future research should expand the research site and sample, to render more generalizable findings. Lastly, the path analysis models showed relatively low R^2^s, suggesting limited predictive effects of the independent variables (Zhu, 2012). Therefore, the results should be interpreted with caution.

The findings on college students indicate the importance of fostering both PAF knowledge and PPL for health-related physical fitness promotion. As emerging adults, college students are less active than children and adolescents but more active than older adult populations, which positions them at the critical stage to adopt the active and healthy lifestyles. As college students spend most of their time living on campus, colleges and universities should create on-campus opportunities for them to build physical capabilities. Elective physical activity classes offer prime opportunities for them to acquire the knowledge, skill, and disposition to be active and improve fitness. Teachers of these physical activity programs should educate students to not only understand physical activity and fitness, but also apply and transfer the learning to sustain their physical activity participation and fitness. In addition, colleges and universities may consider offering students both online and in-person pedagogical or advisory workshops to help them identify and understand their own fitness, skills, and knowledge, which will collectively facilitate students’ disposition to reap the maximal benefits of physical activity for health, wellbeing, and performance.

## Conclusion

PAF knowledge should be incorporated into college physical activity programs as a focused learning content to cultivate students’ literacy and habit so they can receive the health benefits through sports and exercises. To support college students’ fitness development, tailored pedagogical interventions on campus are needed to increase college students’ physical literacy, physical activity, and fitness. Future studies may consider examining possible moderators and mediators (e.g., physical activity behavior) when testing the predictive effects of PPL and PAF knowledge on fitness. As more research in this area emerges, peer researchers should strive to develop and establish an insightful conceptual model to guide future research and practice.

## Data availability statement

The raw data supporting the conclusions of this article will be made available by the authors, without undue reservation.

## Ethics statement

The studies involving humans were approved by Ethics Committee of Wuhan Sports University. The studies were conducted in accordance with the local legislation and institutional requirements. The participants provided their written informed consent to participate in this study.

## Author contributions

BL: Conceptualization, Formal analysis, Investigation, Supervision, Validation, Writing – original draft. YL: Funding acquisition, Investigation, Methodology, Software, Supervision, Validation, Writing – original draft. SC: Investigation, Supervision, Validation, Writing – review & editing. YY: Supervision, Writing – review & editing. ST: Supervision, Writing – review & editing. JY: Supervision, Writing – review & editing. YW: Supervision, Writing – review & editing.
